# Fungal volatile compounds induce production of the secondary metabolite Sodorifen in *Serratia plymuthica* PRI-2C

**DOI:** 10.1038/s41598-017-00893-3

**Published:** 2017-04-13

**Authors:** Ruth Schmidt, Victor de Jager, Daniela Zühlke, Christian Wolff, Jörg Bernhardt, Katarina Cankar, Jules Beekwilder, Wilfred van Ijcken, Frank Sleutels, Wietse de Boer, Katharina Riedel, Paolina Garbeva

**Affiliations:** 1 Netherlands Institute of Ecology (NIOO-KNAW), Department of Microbial Ecology, 6700 AB Wageningen, The Netherlands; 2 Department of Soil Quality, Wageningen University, 6700 AA Wageningen, The Netherlands; 3 Business Unit Bioscience, Wageningen Plant Research, Wageningen University & Research, 6700 AA Wageningen, The Netherlands; 4 Institute of Microbiology, University of Greifswald, 17487 Greifswald, Germany; 5 Center for Biomics, Erasmus Medical Center, 3015 CN Rotterdam, The Netherlands

## Abstract

The ability of bacteria and fungi to communicate with each other is a remarkable aspect of the microbial world. It is recognized that volatile organic compounds (VOCs) act as communication signals, however the molecular responses by bacteria to fungal VOCs remain unknown. Here we perform transcriptomics and proteomics analyses of *Serratia plymuthica* PRI-2C exposed to VOCs emitted by the fungal pathogen *Fusarium culmorum*. We find that the bacterium responds to fungal VOCs with changes in gene and protein expression related to motility, signal transduction, energy metabolism, cell envelope biogenesis, and secondary metabolite production. Metabolomic analysis of the bacterium exposed to the fungal VOCs, gene cluster comparison, and heterologous co-expression of a terpene synthase and a methyltransferase revealed the production of the unusual terpene sodorifen in response to fungal VOCs. These results strongly suggest that VOCs are not only a metabolic waste but important compounds in the long-distance communication between fungi and bacteria.

## Introduction

Interactions and communication among organisms are central to understanding any ecosystem. The essential role of volatile organic compounds (VOCs) in the communication with other organisms, also known as infochemicals, has been acknowledged for more than 30 years^1^. However, their ecological functions have been mainly studied for aboveground plant-plant and plant-insect interactions^2, 3^. In recent years, VOCs are becoming increasingly important in the field of microbial ecology. Due to their unique nature (low molecular mass, high vapor pressure, low boiling point and a lipophilic moiety) VOCs play important roles in the long-distance interactions and communication within the microbial world^4^. In soil and in the rhizosphere, VOCs readily diffuse under atmospheric pressure and travel throughout the abundant air- and liquid-filled pockets of the soil^4, 5^.

Several soil-associated bacteria were shown to have positive effects on plant growth and resistance^6–8^, as well as to have the ability to control plant diseases via production of VOCs^9^. Various studies have documented that VOCs can have diverse roles in the interaction between physically separated microorganisms, ranging from infochemical molecules affecting the behavior, population dynamics and gene expression in responding microorganisms to interference competition tools suppressing or eliminating potential enemies^4, 10–13^. Interestingly, most studies have only examined the role of bacterial VOCs and their effect on plants and fungi. However, the role and function of fungal VOCs on bacteria remains largely unknown. Only few studies demonstrated that the growth of some bacterial species was suppressed by fungal VOCs^14^. For example, VOCs produced by the oyster mushroom *Pleurotus ostreatus* showed inhibitory effects on *Bacillus cereus* and *Bacillus subtilis* ^15^. Another study by Lutz *et al*.^16^ demonstrated that VOCs emitted by *T. atroviride* increased the expression of a biocontrol gene (*phlA*) of *P. fluorescens*.

Previously, we investigated the effect of fungal VOCs on the behavior of phylogenetically different soil bacteria^17^. In these experiments we showed that VOCs emitted by several fungi lead to phenotypical responses in bacteria, for example, by inducing a change in bacterial motility^17^. We observed that the plant pathogenic fungus *Fusarium culmorum* produced a unique cluster of VOCs, consisting primarily of terpenes. When exposed to the VOCs emitted by this fungus, the rhizobacterium *Serratia plymuthica* PRI-2C responded with an induction of motility.

It is plausible that in soil, microorganisms sense changes in their environments via shifts in VOCs blends and adapt their behavior accordingly^11^. Although several studies indicated that VOCs can be used as signaling molecules in microbial inter-species interactions, the following questions remain unanswered: how are VOCs perceived as signals by the interacting microorganism and which regulatory genes and pathways are involved in the response?

To answer these questions, the rhizosphere isolate *S. plymuthica* PRI-2C was grown exposed or unexposed to VOCs emitted by *F. culmorum* and the bacterial transcriptome and proteome were analyzed under each situation to identify the molecular basis of the bacterial response to fungal VOCs. Further, metabolomics analysis and heterologous expression studies were performed to confirm the production of an unusual terpene compound involved in this bacterial-fungal conversation.

## Methods

### Strains and growth conditions

Based on previous work^17^ *S. plymuthica* PRI-2C and *F. culmorum* PV were selected for this study. The bacterial strain *S. plymuthica* PRI-2C was isolated from maize rhizosphere in the Netherlands^18^, pre-cultured from frozen glycerol stocks on 0.1 Tryptic Soy Broth plates (TSB)^19^ and grown for 3 days at 20 °C before use. The fungal strain *F. culmorum* PV was isolated from a sandy dune soil in the Netherlands^20^, pre-cultured on 0.5 Potato Dextrose Agar plates (PDA)^21^ and incubated for 6 days at 20 °C before use.

### *S. plymuthica* PRI-2C genome sequencing

Genomic DNA was extracted from *S. plymuthica* PRI-2C cells using the Qiagen MagAttract HMW DNA kit (Qiagen) and subjected to PacBio RS II sequencing at the Institute for Genome Sciences (IGS), Baltimore, Maryland, USA. PacBio RS II sequences were obtained from 1 SMRT cell. Sequences were filtered using SMRT Analysis server v2.3.0 with default settings and assembled using the RS_HGAP Assembly.3 (HGAP3) protocol, followed by a final Quiver correction using the RS_Resequencing protocol. The Illumina reads obtained from RNA-sequencing were filtered using Fastq MCF^22^ with default settings and aligned against the contigs using BWA V0.7.12^23^. The aligned reads were re-aligned with GATK V3.5.0^24^ to ensure correct indel calls. Contigs were corrected using the re-aligned reads with Pilon^25^. Singular contigs were checked with a custom script and overlapping ends were trimmed. The final circular contig was rearranged to start at the *dnaA* gene in the forward direction. The sequences were annotated using Prokka 1.11^26^. The accession number of the genome after submission to NCBI is CP015613.

### Interaction experiment between *S. plymuthica* PRI-2C and *F. culmorum* PV

**Figure 1:**
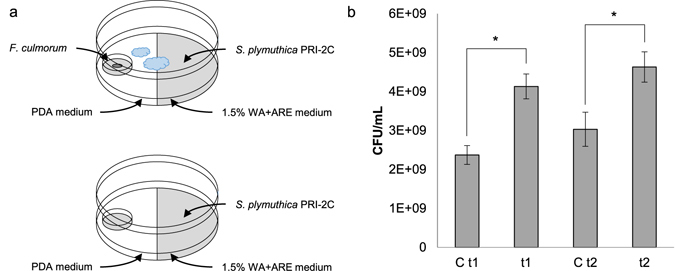
Overview of experimental setup and fungal VOC effect on bacterial growth. Schematic overview of the plate-within-a-plate system (**a**). Growth of *S. plymuthica* PRI-2C exposed to fungal VOCs after 48 h (t1) and 72 h (t2) (**b**). Controls *S. plymuthica* PRI-2C without exposure to VOCs after 48 h (C t1) and 72 h (C t2). Error bars represent standard deviations between replicates (*n* = 3). Asterisk indicates statistical significance (*p* < 0.05) of each time point compared to the respective control.

To investigate the response of *S. plymuthica* PRI-2C to volatiles emitted by *F. culmorum* PV, a plate-within-a-plate system was used (Fig. 1a). *F. culmorum* PV plugs (6 mm in diameter) were inoculated on a 0.5 PDA Petri dish (3.5 cm) that was placed into the partitioned Petri dish (9 cm) and incubated for 3 days at 20 °C. On day 3 *S. plymuthica* PRI-2C inoculum, consisting of 50 μl washed cell suspension in a 10 mM phosphate buffer (pH 6.5) and containing 10^7^ cells/ml, was spread on 1.5% water agar supplied with artificial root exudates (WA + ARE)^17^ of the partitioned Petri dish. As control *S. plymuthica* PRI-2C was exposed to 0.5 PDA medium only. Plates were incubated at 20 °C. Total RNA and cytosolic proteins were extracted after 48 h (time point 1) and 72 h (time point 2). All experiments were performed in triplicates.

To test the effect of fungal VOCs on the growth of *S. plymuthica* PRI-2C, the CFU/ml were determined after 48 h and 72 h (Fig. 1b). After scratching off the cells from the surface dilution series of each treatment were prepared in 10 mM phosphate buffer (pH 6.5). A volume of 100 µl of each serial dilution was plated on 1/10th TSB plates in duplicates. The plates were incubated for three days at 20 °C. Bacterial enumeration was carried out on an aCOlyte Colony Counter (Meintrup DWS Laborgeräte GmbH). A two-tailed tailed Student’s *t*-test (p < 0.05) was used to evaluate the differences between two groups using SPSS (SPSS, Chicago, IL, USA). Results are shown as mean ± S.D. Sample number (*n*) represents biological replicates.

### RNA Extraction

*S. plymuthica* PRI-2C cells were suspended in 2 ml of sterile phosphate buffer and transferred to a double volume of RNAprotect Bacteria Reagent (Qiagen). The mixture was centrifuged at 14,000 rpm for 1.5 min at 4 °C. The supernatant was discarded and the cell pellets were directly frozen in liquid N_2_ and stored at −80 °C until RNA extraction. RNA was extracted using the Aurum Total RNA Mini Kit (Bio-Rad), following the manufacturer’s recommendations. Contaminating DNA was removed using the TURBO DNA-free Kit (Ambion). RNA concentrations and quality were measured on a NanoDrop Spectrophotometer (Isogen Life Science) and on a Fragmentanalyzer RNA 6000 Pico Chip (Agilent).

### RNA sequencing and differential expression analysis

RNA sequencing was performed at Erasmus Center for Biomics, The Netherlands. RNA was depleted of rRNA using the Ribo-Zero rRNA Removal Bacteria Kit (Illumina). A sequencing library was constructed using the KAPA Stranded RNA-Seq Kit (Kapa Biosystems), and sequenced according to the Illumina TruSeq V3 protocol on the HiSeq2000 with a single read 50 bp and 7 bp index.

The obtained Illumina reads from the RNA-sequencing were filtered using Fastq MCF and aligned against the cDNA sequences of *S. plymythica* PRI-2C using Bowtie 2 (2.2.5)^27^ with the following settings: –no-mixed –no-discordant –gbar 1000 –end-to-end. Transcript abundance was estimated using RSEM V1.1.26^28^ and differential expression between the treatments was analyzed using edgeR V3.2^29, 30^. Differentially expressed genes (DEGs) were defined with a cutoff at log fold change of greater than 0.585 or less than −0.585, an FDR of less than 0.05. Data were filtered with a *p*-value of 0.001 (Table S2). Venn diagrams were produced using Venny^31^. Functional prediction and assignment of genes to TIGRFAMs^32^ was accomplished by the in-house developed analysis pipeline ‘Prophane 2.0′ (http://www.prophane.de; Schneider *et al*.^33^. Voronoi treemaps were generated using Paver (Decodon, Greifswald, Germany, http://www.decodon.com/).

### Validation of RNA sequencing data

Validation of RNA sequencing data was performed by Quantitative Real-Time PCR (qRT-PCR) analysis of a subset of differentially expressed genes related to terpene production, motility and a gene encoding for isocitrate lyase. Two housekeeping genes, *rpoB* and *gyrB*, were used as internal standards (Table 1). All experiments were performed in triplicates.

**Table 1:** Primers used in qPCR.

Primer code	Sequence (5′–3′) of primers	target	reference
011535 _F	TGGACCGCATGACAAAGGAG	Putative terpene synthase	this study
011535 _R	TGAAGCGGTATAAGCCAGCC	Putative terpene synthase	this study
015265 _F	TGTCTCAGGTTGACTCGCTG	Flagellin	this study
015265 _R	AGTCGGCATCCTGAATACGG	Flagellin	this study
015275 _F	AGTGGCGTAATGAGTGCCAG	Flagellar protein FliS	this study
015275 _R	ATATAGCCAGCCCTTTGCCG	Flagellar protein FliS	this study
011530 _F	GCGCAATGGAAGAAGATCCG	Arabinose operon regulatory protein	this study
011530 _R	GATTGCCGGTGATGAACTGAC	Arabinose operon regulatory protein	this study
023425 _F	TACACCGATTTCTTCCTGCCG	Isocitrate lyase	this study
023425 _R	TCGCCTTCATCAGTTCGTAGG	Isocitrate lyase	this study
001325 _F	GCCGAAAGGTGAAACCCAAC	DNA-directed RNA polymerase subunit beta RpoB	this study
001325 _R	TGCACGTCGATAACCGTACC	DNA-directed RNA polymerase subunit beta RpoB	this study
000020_F	GCACTACAGCGTGCAGAAAC	DNA gyrase subunit B GyrB	this study
000020_R	GGGTTGGCTGGTAGATCAGG	DNA gyrase subunit B GyrB	this study

First strand cDNA was synthesized from 2 μg previously extracted total RNA with random hexamer primers using the SuperScript VILO cDNA Synthesis Kit (Invitrogen) according to the manufacturer’s protocol. A volume of 4 μl cDNA of each treatment was subjected to qRT-PCR using QuantiNova SYBR Green I PCR master mix (Qiagen) a Qiagen Research Rotor- Gene Q thermal cycler (Qiagen) with the following conditions: initial cycle 95 °C for 2 min, followed by 40 cycles of 95 °C for 10 sec, and 60 °C for 30 sec. Standard curves were established for each cDNA sample to calculate the expression values (Ct-value). Log fold changes in gene expression were determined using the ΔΔCt method^34^, data were normalized against the two housekeeping genes (*rpoB* and *gyrB*), and compared to log fold changes obtained from RNA sequencing data (Figure S1).

### Protein extraction and MS sample preparation

*S. plymuthica* PRI-2C cells were suspended in 2 ml of sterile phosphate buffer and centrifuged at 14,000 rpm for 1.5 min at 4 °C. Cytosolic proteins were extracted using a bead-beating protocol. Fractions were transferred into 2 ml Eppendorf tubes with 0.25 ml of 0.1 mm glass beads (BioSpec Products) and placed into FastPrep-24 lyser tubes (MP Biomedicals). Eight rounds of 30 s lysis at 6.5 m s−1 followed by 5 min incubation on ice were used to lyse the cells. Afterwards, cell debris was removed by centrifugation; determination of cytosolic protein concentration was done using Roti^®^-Nanoquant (Roth). Reduction of disulfide bonds with TCEP, alkylation of thiolgroups with iodoacetamide and subsequent in-solution digestion of proteins using trypsin was performed as described by Muntel *et al*.^35^. Desalting of peptides prior to mass spectrometry analysis using Stage tips was done according to the protocol by Rappsilber *et al*.^36^.

### Setup for label-free protein quantification (LC-IMS^E^) and data analysis

The nanoACQUITY^TM^ UPLC^TM^ system (Waters) was used to separate and to introduce peptides into the Synapt G2 (Waters) mass spectrometer. Parameters for liquid chromatography and IMS^E^ (MS^E^ with ion mobility separation) were used as described previously^37, 38^.

LC-IMS^E^ data were processed using PLGS v3.0.1 Processing parameters were set as follows: Chromatographic peak width and MS TOF resolution were set to automatic, lock mass charge 2 set to 785.8426 Da/e with a lock mass window of 0.25 Da, low energy threshold 200.0 counts, elevated energy threshold 20.0 counts, intensity threshold 750 counts. The data were searched against a randomized *S. plymuthica* PRI-2C database (version February 2016) with added laboratory contaminants and yeast ADH1 sequence (10,090 entries). For positive protein identification the following criteria had to be met: 1 fragment ion matched per peptide, 5 fragment ions matched per protein, 1 peptide matched per protein; 2 missed cleavages allowed, primary digest reagent: trypsin, fixed modification: carbamidomethylation C (+57.0215), variable modifications: deamidation N, Q (+0.9840), oxidation M (+15.9949), pyrrolidonecarboxylacid N-TERM (−27.9949). The protein false discovery rate (FDR) was set to 5%. For the final analysis only identifications based on at least two peptides were considered. In total 9 MS-runs per time point were conducted (3 biological replicates with 3 technical replicates each). For positive identification and reliable quantification, a replicate filter of 4/9 was applied. This reduced the false discovery rate per sample to less than 0.7%. Absolute protein quantification of identified proteins was performed using top3 peptide intensity with spiked-in yeast alcohol dehydrogenase (Waters; concentration of 50 fmol/µl sample) as a reference (Hi3 approach, Silva *et al*.^39^). Data were corrected for detector saturation effects by implementing a correction factor as recently described^38^. To allow comparison between samples absolute protein amounts calculated by PLGS (given in fmol) were normalized (fmol/ng total protein).

Statistical analysis was done using MeV v4.8.1^40^ for proteins that were present in at least four out of nine replicates. Hierarchical clustering and Student’s t-test were performed with the following settings: unequal group variances were assumed (Welch approximation), *p*-values based on all permutation with *p* = 0.01, significance determined by adjusted Bonferroni correction. Only proteins showing at least 1.5 fold changes in addition to statistical significance were considered for further analysis. So-called ‘off/on’ proteins needed to be detected in at least four replicates of one treatment and absent from all replicates of another treatment.

Functional prediction and assignment of proteins to TIGRFAMs^32^, respectively, was accomplished by the in-house developed analysis pipeline ‘Prophane 2.0’ (http://www.prophane.de; Schneider *et al*.^33^. Voronoi treemaps were generated using Paver (Decodon, Greifswald, Germany, http://www.decodon.com/). The mass spectrometry proteomics data have been deposited in the ProteomeXchange Consortium via the PRIDE partner repository^41^ with the dataset identifier PXD004819.

### Volatile trapping

For the collection of volatiles produced during the interaction, partitioned glass Petri dishes with lids connected to a steel trap containing 150 mg Tenax TA and 150 mg Carbopack B (Markes International Ltd., Llantrisant, UK) were used^42^. *F. culmorum* PV was grown on 0.5 PDA at 20 °C for 3 days. On day 3 *S. plymuthica* PRI-2C inoculum, consisting of 50 μl washed cell suspension in a 10 mM phosphate buffer (pH 6.5) and containing 10^7^ cells/ml, was spread on 1.5% WA + ARE. As control *S. plymuthica* PRI-2C was exposed to 0.5 PDA medium only. Plates were incubated for 48 h and 72 h at 20 °C. Volatiles were collected during 48 h and 72 h of incubation at 20 °C. All experiments were performed in triplicates. Traps were removed, capped and stored at 4 °C until analysis using GC-Q-TOF.

The trapped VOCs were desorbed from the traps using an automated thermo desorption unit (Unity TD-100, Markes International Ltd., Llantrisant, UK) at 210 °C for 12 min (He flow 50 ml/min) and trapped on a cold trap at −10 °C. The trapped volatiles were introduced into the GC-Q-TOF (model Agilent 7890B GC and the Agilent 7200 A QTOF, Santa Clara, USA) by heating the cold trap for 3 min to 280 °C with split ratio set to 1:20. The column used was a 30 mm × 0.25 mm ID RXI-5MS, film thickness 0.25 μm (Restek 13424-6850, Bellefonte, PA, USA). VOCs were detected by the MS operating at 70 eV in EI mode. Mass spectra were acquired in full scan mode (30–400 amu, 4 scans/s). MassHunter Qualitative Analysis Software V B.06.00 Build 6.0.633.0 (Agilent Technologies, Santa Clara, CA, USA) was used to control the instrument and for data acquisition and analysis. Mass-spectra were extracted with MassHunter Qualitative Analysis Software V B.06.00 Build 6.0.633.0 (Agilent Technologies, Santa Clara, USA) and compared with the data of the NIST 2014 V2.20 library and the spectrum of sodorifen published in ref. 43.

### Production of sodorifen in *Escherichia coli*

PCR-generated DNA encompassing the complete coding sequence of the sodorifen synthase (SpSS, Q5A_011535) and the methyltransferase (SpMT, Q5A_011540) of *S. plymuthica* PRI-2C were inserted into MCS1 (BamHI, NotI) and MCS2 (NdeI, KpnI) cloning sites of the expression vector pACYCDuet-1 (Novagen, Cm^R^), respectively. The insertion was confirmed by colony PCR and sequencing. The constructed plasmids, pACYCDuet SpSS, pACYCDuet SpMT, pACYCDuet SpSS/SpMT and pACYCDuet empty, were transformed into chemically competent *E. coli* BL21 DE3 carrying the plasmid pMEV, which is a kanamycin-resistant version of the mevalonate pathway-expressing plasmid i-pBbA5c-MevT(CO)-MBIS(CO)-NptII^44^ (Addgene, Peralta-Yahya *et al*.^45^), which was kindly supplied by Thamara Hendricks. Transformants were plated on LB plates supplemented with kanamycin (50 µg/ml), chloramphenicol (50 µg/ml) and 1% glucose. A starter culture of all 4 clones was grown overnight in 5 ml LB medium supplemented with kanamycin (50 µg/ml), chloramphenicol (50 µg/ml) and 1% glucose at 37 °C, 250 rpm. 500 µl of the starter culture was inoculated into 50 ml LB supplemented with kanamycin (50 µg/ml) and chloramphenicol (50 µg/ml) in 250 ml Erlenmeyer flasks. The culture was incubated at 37 °C, 250 rpm until the OD_600_ reached 0.4. At this point 100 µl of each culture were spread on a glass Petri dish with 20 ml LB agar supplemented with kanamycin (50 µg/ml), chloramphenicol (50 µg/ml) and 0.1 M IPTG. Steel trap containing 150 mg Tenax TA and 150 mg Carbopack B were added into the lids and the plates were incubated at 25 °C for 48 h. All experiments were performed in duplicates. Traps were removed, capped and stored at 4 °C until analysis by using GC-Q-TOF. The traps were measured as described in the previous section with a split ratio set to 1:80. Mass-spectra were extracted with MassHunter Qualitative Analysis Software V B.06.00 Build 6.0.633.0 (Agilent Technologies, Santa Clara, USA) and compared with the data of the NIST 2014 V2.20 library and the spectrum of sodorifen published in von Reuss *et al*.^43^ and Domik *et al*.^46^.

## Results

### Genomic features of *S. plymuthica* PRI-2C

The complete genome of *S. plymuthica* PRI-2C was sequenced to facilitate the interpretation of proteomics and transcriptomics data and to gain better insights into the bacterial perception and response to fungal VOCs. The complete genome of *S. plymuthica* PRI-2C is 5,464,425 bp in size. Annotation of the genome predicted 5,002 protein-coding genes, 88 tRNAs and 22 rRNAs (Accession number CP015613).

*S. plymuthica* PRI-2C harbors several gene clusters encoding for secondary metabolites. Based on *in silico* prediction using antiSMASH a total of 36 secondary metabolite gene clusters were identified. One of those gene clusters belonged to the class of terpenes, one to the class of arylpolyene-siderophore, one to the class of butyrolactones, one to nonribosomal peptide synthetase (nrps) -Type I polyketide synthase (t1pks), one to homoserine lactone (hserlactones), one to others, two to nonribosomal peptide synthetase (nrps). Out of the remaining 28 gene clusters, four were identified as putative fatty acids, four as putative saccharides and twenty as non-identified putative gene clusters.

Important for microbial interactions and communication are signal transduction systems that enable bacteria to detect and respond to changes in the environment (Stock *et al*.^47^). We investigated the genome for signal transduction systems using the MIST2 database^48^. The genome of *S. plymuthica* PRI-2C encodes 428 one-component systems (1CSs) and 69 two-component systems (TCSs). Two extracytoplasmic function (ECF) sigma factors were found, which comprise the largest group among the σ70 family^49^. Additionally, 8 genes involved in chemotaxis systems were found (Table S1).

### Effect of fungal VOCs on the growth of *S. plymuthica* PRI-2C

After 48 h of exposure to fungal VOCs the cell density of *S. plymuthica* PRI-2C was 4.13 × 10^9^ CFU/ml and after 72 h of exposure 4.63 × 10^9^ CFU/ml. We observed a significant increase in the growth of *S. plymuthica* PRI-2C exposed to VOCs after both time points as compared to *S. plymuthica* PRI-2C without exposure to VOCs (Fig. 1b).

### Global changes in the transcriptome and proteome of *S. plymuthica* PRI-2C in response to fungal VOCs

**Figure 2:**
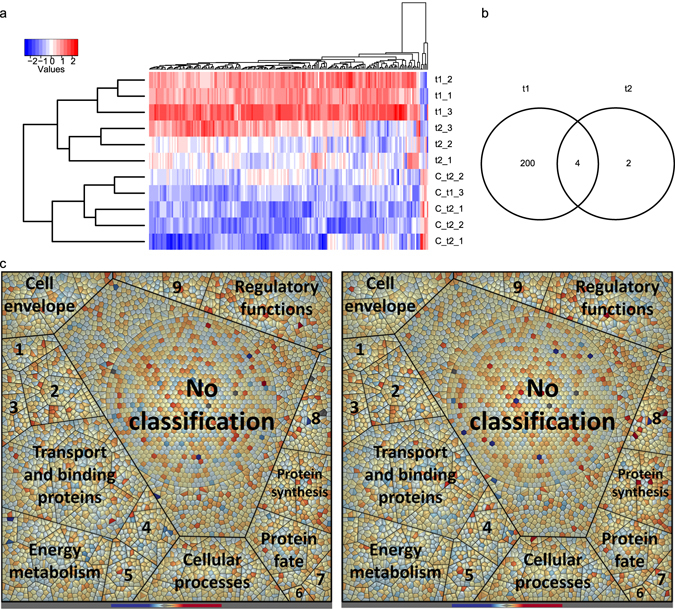
Changes in transcriptome of *S. plymuthica* PRI-2C in response to fungal VOCs. Heatmap of 206 DEGs with at least +/−1.5-fold changes and a p-value less than 0.001 as a cut-off (**a**). Hierarchical clustering was performed using the Pearson Correlation metric. Venn diagram of uniquely and commonly expressed DEGs at t1 and t2 (**b**). Voronoi treemaps visualizing changes in the transcriptome of *S. plymuthica* PRI-2C exposed to fungal VOCs at t1 (C left) and at t2 (C right) (**c**). Functional classification of genes was carried out by Prophane 2.0 and is based on TIGRFAMS. Red color indicates higher expression of the respective gene and blue color indicates lower expression as compared to the control. Each cell represents a single gene; functional classes are separated by thicker black lines. Numbers indicate the following classes: 1 - Fatty acid and phospholipid metabolism, 2 - Biosynthesis of cofactors, prosthetic groups, and carriers, 3 - Purines, pyrimidines, nucleosides, and nucleotides, 4 - Amino acid biosynthesis, 5 - Central intermediary metabolism, 6 - Mobile and extrachromosomal element functions, 7 - Transcription, 8 - DNA metabolism, 9 - Signal transduction.

To study the response of *S. plymuthica* PRI-2C to fungal VOCs, we investigated changes at the transcriptome and proteome level after 48 h (t1) and 72 h (t2) of exposure to *F. culmorum* VOCs. After 48 h of exposure to fungal VOCs 204 differentially expressed genes (DEGs) were identified (199 up- and 5 down-regulated), while after 72 h of exposure only 6 DEGs were detected (5 up- and 1 down-regulated) (Fig. 2a, Table S2). Only 2 DEGs were unique for t2 and 4 DEGs were expressed at both times points (Fig. 2b). These 4 genes were Q5A_006040 (coding for the Pyrimidine-specific ribonucleoside hydrolase RihA), Q5A_018030 (coding for the putative HTH-type transcriptional regulator YahB), Q5A_003275 (coding for the UDP-N-acetylmuramoyl-L-alanyl-D-glutamate–2,6-diaminopimelate ligase) and Q5A_011375 (coding for a hypothetical protein). Most of the DEGs at t1 were assigned to functions involved in energy metabolism, transport and binding proteins, regulatory functions, cell envelope, cellular processes, protein synthesis and fate as well as unknown function. For t2 DEGs were assigned to functions involved in DNA metabolism, regulatory functions, cell envelope and unknown function (Fig. 2c, Table S2).

**Figure 3:**
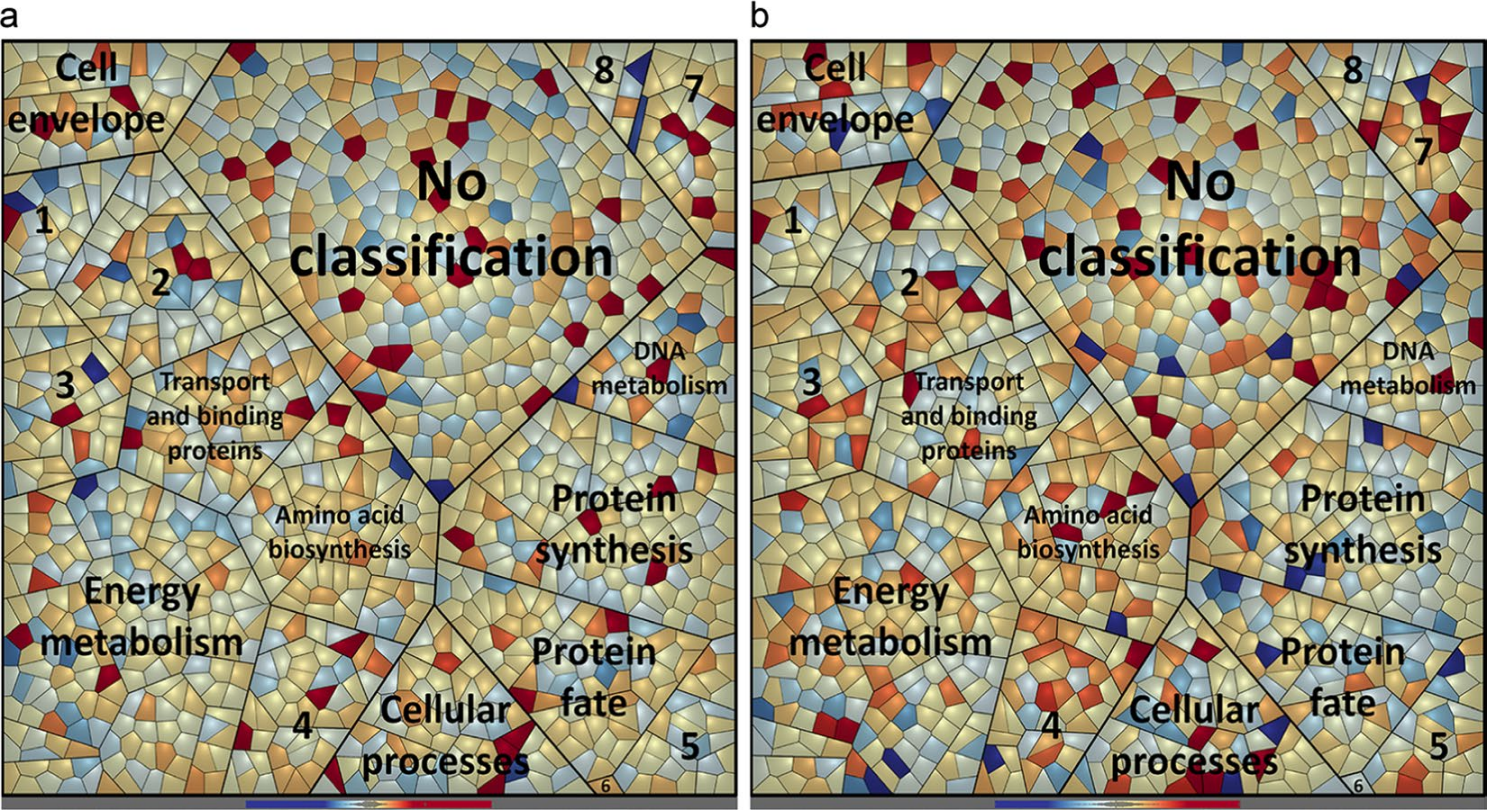
Voronoi treemaps visualizing changes in the proteome composition of *S. plymuthica* PRI-2C exposed to fungal VOCs. Quantitative expression values of *S. plymuthica* PRI-2C proteins at t1 of exposure (**a**) and at t2 (**b**). Functional classification of proteins was carried out by Prophane 2.0 and is based on TIGRFAMS. Red color indicates higher expression of the respective protein and blue color indicates lower expression as compared to the control. Each cell represents a single protein. Functional classes are separated by thicker black lines. Numbers indicate the following classes: 1 - Fatty acid and phospholipid metabolism, 2 - Biosynthesis of cofactors, prosthetic groups, and carriers, 3 - Purines, pyrimidines, nucleosides, and nucleotides, 4 - Central intermediary metabolism, 5 - Transcription, 6 - Mobile and extrachromosomal element functions, 7 - Regulatory functions, 8 - Signal transduction.

On the proteome level absolute amounts for a total of 1,574 proteins were obtained by applying the LC-MSE approach^39^ (Table S3). A large number of these proteins were assigned to functions associated with metabolic processes (584 proteins; e.g. biosynthesis of amino acids and cofactors; fatty acid, energy and central intermediary metabolism) and genetic information processing (311 proteins; e.g. protein synthesis and protein fate, DNA metabolism and transcription). Moreover, a high number of so far uncharacterized or unclassified proteins (448 proteins) were detected (Fig. 3, Table S3).

**Figure 4:**
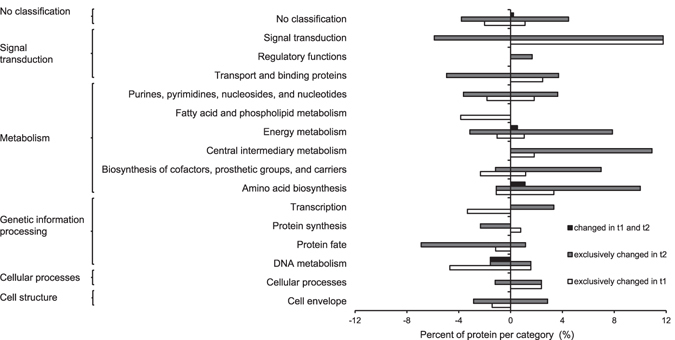
Impact of fungal volatiles on *S. plymuthica* PRI-2C protein content. The percentage of proteins with at least 1.5 fold change in relation to the number of identified proteins is depicted. Black bars indicate proteins with changed amount in both, early (t1, 48 h) and late (t2, 72 h) sampling points, grey bars indicate changed amounts on late sampling point, white bars indicate changed amount in early sampling point. Functional annotation is based on TIGRFAMS.

Longer exposure (72 h) to fungal VOCs led to higher number of proteins with a significantly changed amount as compared to the control. Overall 169 proteins were differentially expressed (significance determined by t-test and expression change of more than 1.5 fold) when exposed to fungal VOCs as compared to the non-exposed control (Table S3). At t1 51 differentially expressed proteins (DEPs) were identified (28 up- and 23 down-regulated) while at t2 124 DEPs were identified (78 up- and 46 down-regulated). For both time points mainly proteins involved in metabolic processes (amino acid biosynthesis, energy and central intermediary metabolism, biosynthesis of cofactors), signal transduction, transport and binding proteins and several uncharacterized proteins were found to be differentially expressed (Fig. 4, Table S3).

### Functional gene and protein categories affected by fungal VOCs

#### Differentially expressed genes and proteins related to chemotaxis and motility

The expression of one gene, Q5A_015275 (coding for the flagellar protein FliS) relating to chemotaxis and motility was up-regulated at t1 when *S. plymuthica* PRI-2C was exposed to fungal VOCs (Table S2). One protein, Q5A_015505 (Methyl-accepting chemotaxis protein IV) was identified at t1 (Table S3) and is known to act as a sensor detecting attractants and promoting bacterial movement towards suitable sites for colonization^50^.

#### Differentially expressed genes and proteins related to signal transduction

29 genes related to signal transduction were up-regulated in our transcriptomics dataset (Table S2). Due to the high number we focus here on genes coding for proteins related to small molecule interactions, protein interactions and two-component systems. Cyclic di-GMP phosphodiesterase YhjH (Q5A_007585) is known to be involved in regulating the levels of c-di-GMP that control cell motility (the flagella) and adhesion (adhesive curli fimbriae)^51^. Phytochrome-like protein Cph2 (Q5A_015970) acts as a photoreceptor that perceives light signals^52^. The putative diguanylate cyclase AdrA (Q5A_005955) is an integral component of the membrane that induces cellulose synthesis, cell adherence to abiotic surfaces and swimming and swarming motility^53^. Serine/threonine-protein kinase HipA (Q5A_018950) can inhibit cell growth and induces multidrug tolerance^54^. Sensor histidine kinase GlrK (Q5A_019275) belongs to a two-component regulatory system that up-regulates transcription of sRNA^55^. The two-component system Nitrogen assimilation regulatory protein (Q5A_025300) was up-regulated in both, transcriptomics and proteomics data at t1. The expression of the corresponding gene is usually activated in response to nitrogen limitation^56^. Nitrate/nitrite response regulator protein NarL (Q5A_015065 and Q5A_018475) is involved in the modulate transcription of genes needed for anaerobic respiration with nitrate or nitrite as electron acceptors^57^. Transcriptional regulatory protein CitB (Q5A_019710) is involved in the expression of citrate-specific fermentation genes^58^.

In the proteomics dataset, the glucose-specific phosphotransferase enzyme IIA component (Q5A_018245) and nitrogen regulatory protein (Q5A_022765) were induced at t1 and t2 respectively (Table S3). Glucose-specific phosphotransferase enzyme IIA component is part of the sugar phosphotransferase system that is involved in glucose transport across the cell membrane^59^. The nitrogen regulatory protein is involved in the regulation of nitrogen utilization^60^. The sensor protein QseC (Q5A_021135) was only identified at t2, which is a member of the two-component regulatory system QseBC that activates the flagella regulon by activating transcription of FlhDC in *E. coli* ^61^. The PTS system fructose-specific EIIBC component (Q5A_017065), which is involved in the translocation of fructose across the membrane (Prior and Kornberg^62^), was repressed at t2.

#### Differentially expressed genes and proteins related to the cell envelope

Five genes coding for proteins related to the cell envelope were up-regulated at t1 of exposure to fungal VOCs (Table S2). The outer membrane protein W (Q5A_013980) is assumed to be involved in the protection of bacteria against various forms of environmental stress^63^. UDP-3-O-(3-hydroxymyristoyl)glucosamine N-acyltransferase (Q5A_019950) is involved in the biosynthesis of lipid A of the outer membrane^64^. Fimbria A protein (Q5A_015665) is a major structural component of mannose-resistant fimbriae^65^ and the putative fimbrial chaperone YfcS (Q5A_021485) is involved in pilus organization. At both t1 and t2, the gene Q5A_003275 (UDP-N-acetylmuramoyl-L-alanyl-D-glutamate–2,6-diaminopimelate ligase) was up-regulated, which is involved in the biosynthesis of bacterial cell-wall peptidoglycan^66^.

At t2 on the proteomics level, two proteins, UDP-N-acetylmuramate–L-alanyl-gamma-D-glutamyl-meso-2,6-diaminoheptandioate ligase (Q5A_001880) and UDP-N-acetylglucosamine 1-carboxyvinyltransferase (Q5A_022690) were present in higher levels. These proteins are involved in the peptidoglycan recycling pathway, part of cell wall biogenesis^67, 68^.

The expression of three proteins was reduced at t1 and t2. The methionine-binding lipoprotein MetQ (Q5A_007015) was reduced at t1 and is involved in the transport of methionine^69^. D-alanyl-D-alanine carboxypeptidase DacA (Q5A_005980) was reduced at t2 and plays a role in the pathway peptidoglycan biosynthesis. Mannose-1-phosphate guanylyltransferase 1 (Q5A_007950) was repressed at t2 and is involved in the synthesis of GDP-alpha-D-mannose.

#### Differentially expressed genes and proteins related to energy metabolism

Three genes, Q5A_001620 (fumarate reductase subunit D), Q5A_011355 (Glutathione S-transferase GST-6.0) and Q5A_024300 (Putative 3-oxopropanoate dehydrogenase) were upregulated at t1 (Table S2). The latter one is involved in the degradation of beta-alanine^70^.

On the transcriptome level, alpha-acetolactate decarboxylase (Q5A_018170) and isocitrate lyase (Q5A_023425) were downregulated at t1, while the latter one was found in higher amounts on the proteome level at t1. Isocitrate lyase catalyzes the bypass of decarboxylation steps of the TCA cycle (glyoxylate shunt). The Glyoxylate shunt is upregulated under different stress conditions, e.g. oxidative stress and antibiotic stress.

#### Differentially expressed genes and proteins related to biosynthesis of natural products

We observed an upregulation of gene expression of Q5A_011535, which encodes a putative terpene synthase, at t1 of exposure to VOCs (Table S2). The corresponding protein showed the highest induction rate of all proteins in the proteomics dataset at t2 (Table S3). This gene is part of a 3-gene cluster comprising genes from the DXP pathway for isoprenoid biosynthesis (DXP synthase Q5A_011545), isopentenyl diphosphate isomerase (Idi, Q5A_011550) and a methyl transferase (Q5A_011540).

### Volatile analysis of *S. plymuthica* PRI-2C and *F. culmorum* PV interaction and heterologous expression of sodorifen

**Figure 5:**
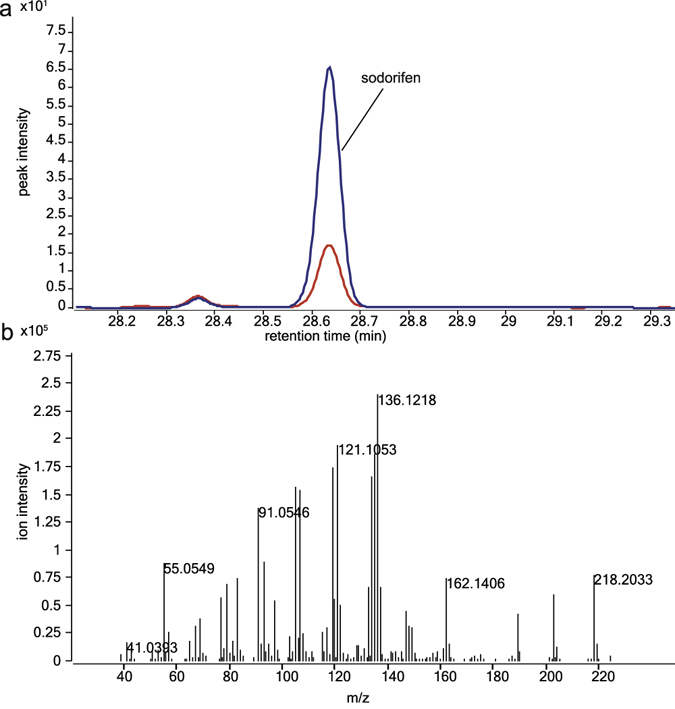
Volatile spectra of the sodorifen peak at RT 28.6 produced by *S. plymuthica* PRI-2C exposed to fungal VOCs (blue) and by *S. plyumthica* without exposure to fungal VOCs (red) (**a**) and mass spectrum of sodorifen produced by *S. plymuthica* PRI-2C (**b**).

As in both, transcriptomics and proteomics datasets, the putative terpene synthase (Q5A_011535) was upregulated in response to *F. culmorum* VOCs we further investigated the production of the terpene compound encoded by this gene. By comparing the VOC emission of S. *plymuthica* PRI-2C exposed to *F. culmorum* VOCs to S. *plymuthica* PRI-2C unexposed to fungal VOCs, we observed one particular compound produced in higher amounts in response to fungal VOCs. This compound was identified as sodorifen (C_16_H_26_; mass = 218.2033; RT = 28.62) (Fig. 5).

**Figure 6:**
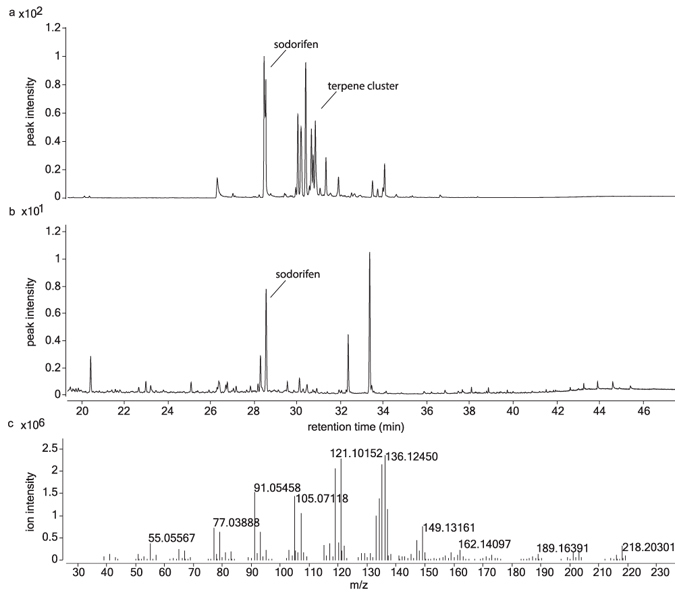
Volatile spectra of *E. coli* producing sodorifen (**a**) in comparison to *S. plymuthica* PRI-2C (**b**) and mass spectrum of sodorifen produced by *E. coli* co- expressing SpSS and SpMT (**c**).

Sodorifen is a recently discovered terpene, which is likely derived from farnesyl pyrophosphate (FPP) but would need an additional methylation to reach its final number of carbons (C16). To confirm that the terpene synthase and methyltransferase can mediate the production of sodorifen, the synthase and methyltransferase genes Q5A_011535 and Q5A_011540 were expressed in an *E. coli* strain overproducing FPP. Upon co-expression of both genes, formation of sodorifen was detected in the headspace of *E. coli* cultures, confirming involvement of these genes in the synthesis of sodorifen (Fig. 6a). No products were detected upon expression of either Q5A_011535 (now called SpSS) or Q5A_011540 (now called SpMT) alone. In addition to sodorifen we observed a cluster of other unidentified terpene compounds following the peak of sodorifen (Fig. 6b). This suggests that the terpene synthase is a multi-product terpene synthase that produces a set of sodorifen like terpenes simultaneously. Indeed a number of these peaks were also found in the headspace of *Serratia* (Fig. 6b).

### Comparison of the sodorifen cluster in other bacterial species

**Figure 7:**
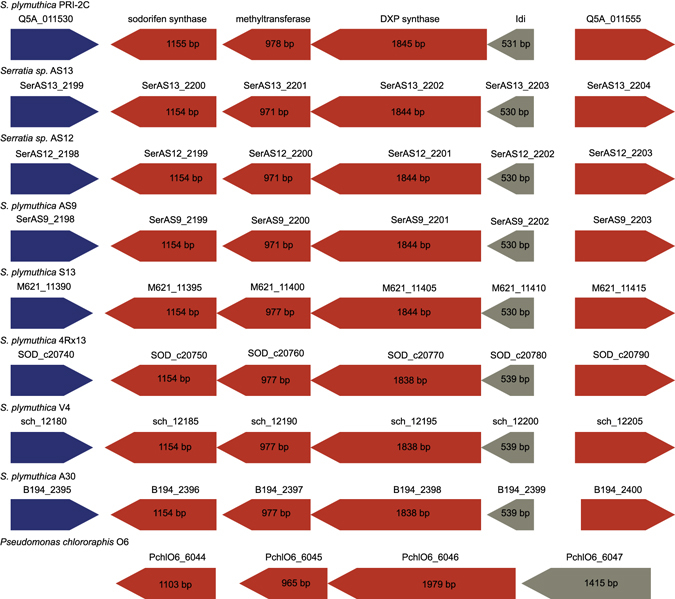
Comparison of the sodorifen cluster within other bacteria. The sodorifen synthase (Q5A_011535) forms a cluster with three additional genes, methyltransferase (Q5A_011540), 1-deoxy-D-xylulose-5-phosphate synthase (Q5A_011545) and isopentenyl-diphosphate delta-isomerase (Q5A_011550), which are positioned in the sense direction within the genome of *S. plymuthica* PRI-2C. The cluster is bordered by genes that are in antisense orientation. Red indicates biosynthesis genes, blue indicates regulatory genes and grey indicates other genes.

We compared the presence of the sodorifen cluster with other bacterial species using antiSMASH^71^. The results show that the sodorifen cluster of *S. plymuthica* PRI-2C is present in several closely related isolates, including the originally described sodorifen producer *S. plymuthica* 4Rx13 (Fig. 7).

## Discussion

**Figure 8:**
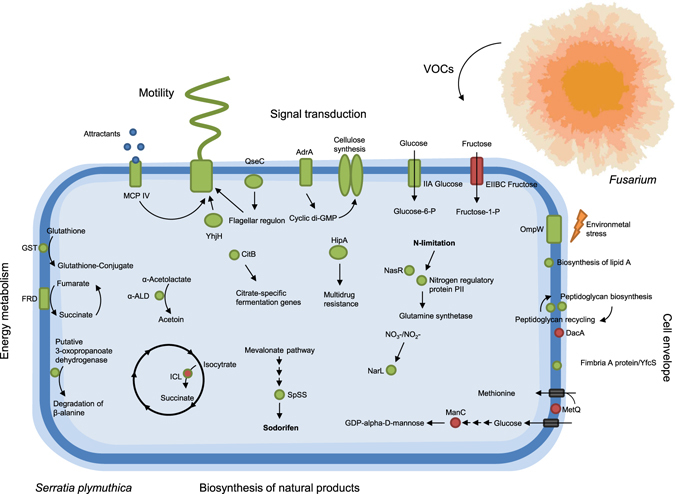
Schematic overview of the most important changes in *S. plymuthica* PRI-2C transcriptome and proteome in response to fungal VOCs. The overview includes genes and proteins involved in major pathways that have been detected in this analysis and genes and proteins whose amounts were either decreased (red) or increased (green) after exposure to fungal VOCs.

The ability of microorganisms to communicate is well recognized, however, deciphering the language and the mechanism of communication remains a challenging task. In the recent years several independent studies, including our own, indicated VOCs as ideal info-chemicals for mediating both, short- and long-distance interactions^4, 10, 11^. Recently, we have shown that VOCs produced by *Fusarium culmorum* affect the motility of the rhizosphere isolate *Serratia plymuthica* without affecting bacterial cell numbers^17^. In the current study we applied both transcriptomic and proteomic approaches to understand the mechanisms underlying bacterial perception and responses to fungal VOCs. Integrated data of our complementary Omics-approach are summarized in Fig. 8.

Access to the complete genomic sequence of an organism is crucial for a reliable interpretation of functional omics data as it enables to correctly identify differentially expressed genes and proteins. Hence, prior to the transcriptomic and proteomic analysis the complete genome of *S. plymuthica* PRI-2C was sequenced and submitted to NCBI. Both transcriptomics and proteomics approaches identified several genes and proteins related to motility, which is in line with the induced *S. plymuthica* motility in response to fungal VOCs. Among these, FliS was described as a flagellin-specific chaperone that prevents premature polymerization of flagellins by facilitating its export^72^. Fimbria A protein was identified in *S. marcescens* as a major structural component of mannose-resistant fimbriae involved in cell-adhesion^65^. On the proteomics level the methyl-accepting chemotaxis protein IV was identified, which acts as chemotactic-signal sensor that detects attractants and promotes bacterial movement towards suitable sites for colonization^50^. Bacterial motility is an important strategy to move toward a favorable stimulus or away from an unfavorable one^73^. We speculate that the increased motility in *S. plymuthica* in response to *F. culmorum* VOCs indicates that fungal VOCs act either as chemo attractants or repellents in these fungal-bacterial interactions.

Next to motility our results revealed several differentially expressed genes and proteins involved in signal transduction. These genes and proteins were involved in the regulation of motility, cell growth, and multidrug tolerance or were part of the two-component system, involved in the regulation of nutrient uptake across the membrane and in the regulation of nutrient limitation. Based on the fact that VOCs modulate antimicrobial activity, Kim *et al*.^12^ hypothesized that VOCs could modulate multidrug resistance. Indeed, the study by Kovač *et al*.^74^ showed that the monoterpene (−)-α-pinene modulates antibiotic resistance in *Campylobacter jejuni* and decreased membrane permeability, which was related to the increased expression of the outer membrane protein product of the *omp50* gene. The authors speculated that the up-regulation of *omp50* is a strategy for bacterial adaptation contributing to decreased membrane permeability and decreased antimicrobial influx. Overexpression of the observed HipA protein has been described to lead to multidrug tolerance in *E. coli* (Coerreia *et al*. 2006). Thus, we suggest that fungal VOCs may as well modulate multidrug tolerance in bacteria.

Some VOCs might serve as energy source under nutrient limited conditions and stimulate the growth of other organisms^11, 75^. Recently Tyc *et al*.^75^ revealed that bacterial response to VOCs can vary from growth stimulation to growth inhibition. In this study, we observed a significant increase in *S. plymuthica* PRI-2C cell numbers when exposed to fungal VOCs. It seems plausible that bacteria take up carbon- or nitrogen-containing volatiles produced by other microorganisms in their surroundings.

In addition we observed a number of upregulated genes and proteins relating to cell envelope biogenesis. Induction of these genes and proteins could imply that the bacterial cells activate membrane biosynthetic pathways when exposed to certain fungal VOCs that causing damages in the bacterial cell wall and thus require repair. Similar findings have been observed by Yung *et al*.^76^ when *E. coli* was exposed to N-methyl-2-pyrrolidone. However, at the same time several genes and proteins relating to cell wall/envelope biogenesis were repressed.

In a previous study we observed that *F. culmorum* emits a cluster of terpenes^17^. The interaction between terpenes and the bacterial cell of pathogenic bacteria wall has been mainly described as increased permeability of the cell membrane leading to a loss of microbial viability^77, 78^. Kovač *et al*.^74^ described a concentration dependent effect of (−)-α-pinene on antimicrobial resistance in *C. jejuni*, leading to either inhibition of antimicrobial efflux in lower concentrations while in higher concentrations leading to increased permeability of the cell membrane thereby promoting the influx of antimicrobials. Our results indicate that terpenes emitted by *F. culmorum* make the cell wall more permeable for VOCs to enter and to be taken up by the cell.

Volatiles may play a role as elicitors that activate gene clusters involved in secondary metabolites production. Likewise, antibiotic production triggered by volatiles mediated interactions was observed in *Pseudomonas aeruginosa* during co-culture with *Enterobacter aerogenes* and in *P. fluorescens* Pf0-1 in response to volatiles produced by *Collimonas pratensis* ^42, 79^.

Moreover, in *Chromobacterium violaceum* and *P. aeruginosa*, several monoterpenes increased violacein and pyocyanin production, respectively^80^. Here we observed an up-regulation of a terpene synthase gene encoding the synthesis of a terpene volatile compound. This compound was identified as sodorifen, an unusual volatile with extraordinary structure, where every carbon atom is substituted with either methyl or methylene group^81^. However, little is known about the biosynthesis of sodorifen other than the study by Domik *et al*.^81^ that reports that a terpene synthase is indispensable for the synthesis of sodorifen and that it descends from the terpene metabolism. Similarly, knockout mutants of the methyl transferase found in the same operon showed no sodorifen production^46^. We confirmed in our work that a terpene synthase and methyltransferase are essential for the biosynthesis of sodorifen by co-expressing SpSS and SpMT in *E. coli*. Furthermore, we demonstrated that methylation of FPP is necessary before the cyclisation by the terpene synthase. This was similarly demonstrated by Wang and Cane^82^ for the biosynthesis of methylisoborneol, a methylated monoterpene with an earthy odor, from *Streptomyces coelicolor*.

The sodorifen synthase from *Serratia* is a multiproduct terpene synthase and sodorifen was produced together with a cluster of other terpene compounds. This is in agreement with the analysis of knockout mutants of either the terpene cyclase or the methyl transferase of the sodorifen operon which lacked the production of sodorifen including a number of other terpenoids^81^. Sodorifen has been described to be produced by several *Serratia* species^83^. Even though *S. plymuthica* PRI-2C was previously reported as a non-producer of sodorifen, this work reveals that the compound is produced under certain conditions and in even higher amounts in response to fungal VOCs. Moreover, in a recent study that described the sodorifen cluster in *S. plymuthica* 4Rx13^81^, the cluster was not detected in *S. plymuthica* PRI-2C when using antiSMASH. This is most likely due to the fact that at the time of the study the genome annotation of this strain was still in draft state and the described cluster could not be found. Comparison of the cluster with the complete genome of *S. plymuthica* PRI-2C shows a very high sequence similarity at the amino acid level of all four genes of the cluster to other *Serratia* strains including *S. plymuthica* 4Rx13.

This work indicates that terpenes may be important molecules in the communication between different microorganisms and hence, may serve as a *lingua franca* in fungal- bacteria interactions.

## Electronic supplementary material

Supplementary Information

Table S1

Table S2

Table S3
