# Supplementary material for: Fungal volatile compounds induce production of the secondary metabolite Sodorifen in Serratia plymuthica PRI-2C
Source: Sci Rep. 2017 Apr 13;7:862. doi: 10.1038/s41598-017-00893-3 (PMC5429845; doi:10.1038/s41598-017-00893-3)
Supplement: Supplementary file 1 — Supplementary Information [file 41598_2017_893_MOESM1_ESM.docx]

**Supplementary Information**

**Fungal volatile compounds induce production of the secondary metabolite Sodorifen in *Serratia plymuthica* PRI-2C**

**Ruth Schmidt^1^, Victor de Jager^1^, Daniela Zühlke^4^, Christian Wolff^4^, Jörg Bernhardt^4^, Katarina Cankar^3^, Jules Beekwilder^3^, Wilfred van Ijcken^5^, Frank Sleutels^5^, Wietse de Boer^1,2^, Katharina Riedel^4^ and Paolina Garbeva*^1^**

^1^Netherlands Institute of Ecology (NIOO-KNAW), Department of Microbial Ecology, 6700 AB Wageningen, The Netherlands

^2^Department of Soil Quality, Wageningen University*,* 6700 AA Wageningen, The Netherlands

^3^Business Unit Bioscience, Wageningen Plant Research, Wageningen University & Research, 6700 AA Wageningen, The Netherlands

^4^Institute of Microbiology, University of Greifswald, 17487 Greifswald, Germany

^5^Center for Biomics, Erasmus Medical Center, 3015 CN Rotterdam, The Netherlands

**Correspondence:**

*Ruth Schmidt and Paolina Garbeva, Netherlands Institute of Ecology (NIOO-KNAW), Department of Microbial Ecology, PO BOX 50, 6700 AB Wageningen, The Netherlands, [r.schmidt@nioo.knaw.nl](mailto:r.schmidt@nioo.knaw.nl) or [p.garbeva@nioo.knaw.nl](mailto:p.garbeva@nioo.knaw.nl)


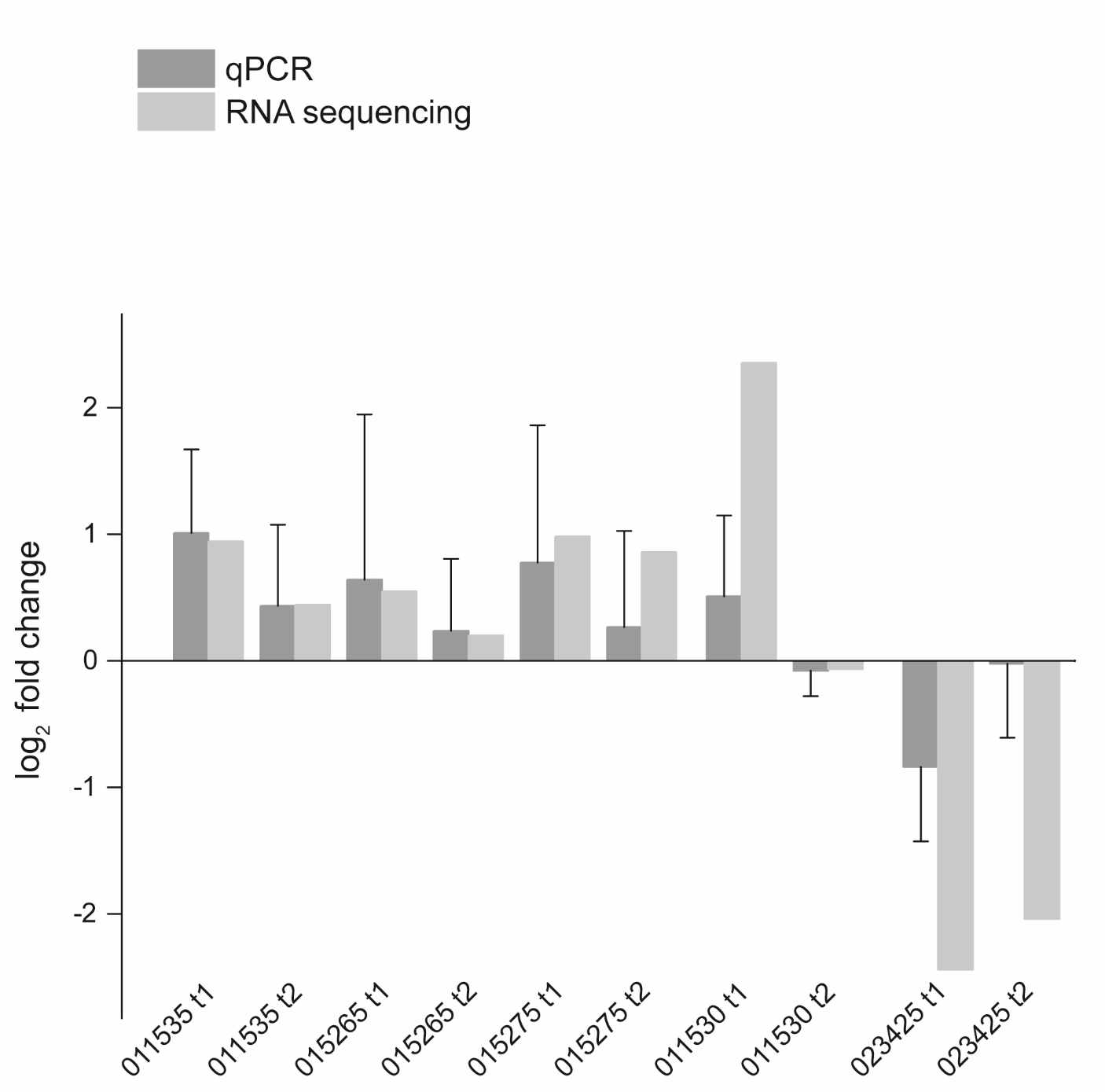


**Figure S1**: qPCR validation of the RNA-seq data at two time points (t1 and t2); 011535 (Putative terpene synthase), 015265 (Flagellin), 015275 (Flagellar protein FliS), 011530 (Arabinose operon regulatory protein), 023425 (Isocitrate lyase). Log_2_ fold changes determined from the relative Ct values of the five genes were compared to those detected by RNA-seq. All Ct values were normalized to two housekeeping genes (rpoB and gyrB). Positive values correspond to higher expression and negative values to lower expression in *S. plymuthica* PRI-2C samples.
